# 

**Published:** 1833

**Authors:** 


### CONTENTS OF NO. XXVII.

### Essays and Cases.

ART. PAGE.

I.—EPIDEMIC CHOLERA in Yucatan. By Henry Perrine, M. D. 321

II. Epidemic Cholera in Columbus, Ohio. By William McClay Awl, M. D. - - - - - - - - - 341

III. Spinal Irritation. By Charles C. Hildreth, M. D. - - 349

IV. Fistula of the Stomach. By Dr. John H. Cook. - - 353

V. Worms in the Bladder. By Dr. Leonard Bardwell. - - 354

VI. Geology of Ohio. By John L. Riddell, A. M. - - - 356

Addendum to the same. By the Editor. - - - - 364

VII. Shooting Stars. By the Editor. - - - - - 368

### Reviews and Bibliographical Notices.

VIII. American Cyclopædia of Practical Medicine and Surgery. Edited by Isaac Hays, M. D. - - - - 386

IX. Imponderable Agents, - - - - - 410

### Miscellaneous Intelligence.

### ANALECTIC.

1. A Sketch of Broussais' Doctrines, drawn up by himself, - 417
2. Death of M. Portal, - - - - - - - 421
3. On Water, applied externally as a remedial agent, - - ib
4. Ulceration of the Stomach, - - - - - 425
5. Dr. Graves on Peritonitis, - - - - 426
6. Chemical Pathology of the Blood in Cholera, - - - 428
7. Treatment of Hooping Cough, - - - - - - - 490
8. Reproduction of the Crystalline Lens after the operation for Cataract, - - - - - - - - ib
9. On the Bearing of Epidemics upon Medical Statistics and Political Economy, - - - - - - - 431
10. New and singular variety of Hermaphroditism, - - 433
11. Extirpation of a degenerated Ovarium, - - - 436
12. Case of Hæmorrhage of the Uterus, arrested by compression of the descending Aorta, - - - - - - 437
13. Professor Weber's experiments on the Sensibility of the Skin, 439
15. Berzelius on the Chemical Constitution of Urine in various diseases, - - - - - - - - 444

### CONTENTS.

16. Observations on Local Blood-letting, and some new methods of practising it, - - - - - - 447
17. On the Oil of the Croton Tiglium, as a Purgative for Children, 449
18. Indian Ophthalmia, treated with success with Alum, - - 450

### ANALYTICAL.

1. Removal of the Clavicle in a state of Osteo-Sarcoma. By Prof. Warren, - - - - - - - 451
2. Injurious administration of the Sulphate of Quinine. By Dr. Monett, - - - - - - - 452
3. Effects of Lightning corrected by the Cold Affusion. By Dr. Young, 456
4. Cholera cured by the Actea Racemosa. By the same, - 557
5. On Fractures of the Thigh and Leg. By Prof. Smith of Baltimore, 558
6. Admission of Air into the Veins, - - - 563

### ORIGINAL.

1. Epidemic Cholera in the Peninsula of Yucatan, - - 565
2. Bad Effects of Animal Diet, - - - - - 566
3. Remedy for Gleet, - - - - - - ib
4. Open Foramen Ovale, - - - - - 567
5. Acephalous Fœtus, living eight hours, - - - - 568
6. Poisoning by Oil of Tansey, - - - - - 569
7 Notice of a suddenly fatal disease, at Stamford, Kentucky, 570
8. Permanent Paralysis of one side of face, from disease about the angle of the Jaw, - - - - - - 473
9. Catalogue of Plants in Kentucky, - - - - 474
10. Cultivation of Botany in Ohio, - - - - 476
11. Cincinnati Medical Society, - - - ib
12. Report of the Commissioners appointed to inquire into the condition of the Medical College, and Commercial Hospital and Lunatic Asylum of Ohio, - - - - - - 477
13. Repeal of the Medical Law of Ohio. Qualified approval. Suggestions about another Law, - - - 479
14. School for the Instruction of the Blind, - - - 483
15. Education Convention of Kentucky, - - 486
16. Introduction of Tropical Plants into Florida, - - ib
17. Winter Residence for Consumptive Patients, - - 487
18. Subsidence of the Cholera in the West. Health of Cincinnati, ib
19. Medical Classes in the West for 1834, - - - 488
20. Medical Obituary, - - - - ib

